# Blunted Post-COVID-19 Humoral Immunity in Patients With CNS Demyelinating Disorders on Anti-CD20 Treatments

**DOI:** 10.3389/fneur.2022.843081

**Published:** 2022-02-23

**Authors:** Kelli M. Money, Ursela Baber, Emma Saart, Soleil Samaan, Jacob A. Sloane

**Affiliations:** Department of Neurology, Beth Israel Deaconess Medical Center, Boston, MA, United States

**Keywords:** multiple sclerosis, disease modifying therapy, COVID-19 antibody, natalizumab, anti-CD20 monoclonal antibodies

## Abstract

With unclear characteristics of post-infection and post-vaccination immunity, the multiple sclerosis community lacks evidence to guide patients on their continued coronavirus disease 2019 (COVID-19) infection risk. As disease modifying treatments all modulate the immune system, we expect their use to alter acquired immunity to COVID-19, but the specific impact of individual treatments is unclear. To address this, we analyzed the patient and COVID-19 specific characteristics associated with post-infection humoral immunity in 58 patients with central nervous system (CNS) demyelinating disorders in the Boston metropolitan area. Univariate analysis of variance was performed using Mann Whitney U test for continuous variables, and Chi Square or Fisher Exact test for nominal variables. Univariate and stepwise multivariate nominal logistic regression identified clinical characteristics associated with COVID-19 specific nucleocapsid IgG antibody formation post-infection. Our cohort demonstrated a 42% post-infection seropositive rate with a significantly higher rate observed with shorter duration between infection and antibody collection and use of natalizumab over no/other treatment. Use of anti-CD20 treatments compared to no/other treatment was associated with a significantly lower rate of seropositivity. However, only shorter duration between infection and antibody collection as well as use of no/other treatment compared to anti-CD20 treatment were found to be independently associated with increased likelihood of post-infection seropositivity. Additionally, we demonstrate durability of antibody response up to 9 months in a small subset of patients. Thus, our data supports that patients with CNS demyelinating disorders regardless of DMT are able to form a measurable antibody response after COVID-19 infection, and that patients on anti-CD20 treatments form less robust immunity after COVID-19 infection.

## Introduction

The evolving coronavirus disease 2019 (COVID-19) pandemic and availability of multiple COVID-19 vaccines have made it clear that the best solution to the COVID-19 pandemic is sustained immunity against the virus. Given the large number of patients with central nervous system (CNS) demyelinating disorders that require immunomodulatory or immunosuppressive medications, identifying the forms and duration of post-infection and post-vaccination immunity in specific subpopulations is critical to guide patient care.

Several studies have demonstrated both humoral and cellular immunity are formed after COVID-19 infection in >90% of the general population, although the duration remains debated (1–4). Patients with multiple sclerosis (MS) and other CNS demyelinating disorders have been found in cohort studies to have an average rate of post-infection seropositivity between 41 and 80% (5–10). Disease modifying therapy (DMT) likely impacts humoral immunity to COVID-19, with small studies suggesting blunted and shorter duration responses in patients on anti-CD20 monoclonal antibodies (5, 7, 9–11). Thus, we currently lack sufficient data to guide patients, especially those that remain unvaccinated, on their likelihood of protective immunity post-infection.

In this study, we evaluated the clinical characteristics associated with COVID-19 IgG seropositivity in a cohort of 58 patients with CNS demyelinating disorders and prior COVID-19 infection. We hypothesized that lymphocyte depleting/sequestering agents would decrease seropositivity.

## Methods

We collected physician reported data via survey from patients with CNS demyelinating disorders treated at Beth Israel Deaconess Medical Center in Boston, MA. Patients were included if they had PCR or antibody confirmed COVID-19 or if COVID-19 was suspected based on typical symptoms, regardless of vaccination status (12, 13). Patients with relapsing remitting MS (RRMS), primary or secondary progressive MS (PPMS and SPMS, respectively), and neuromyelitis optica spectrum disorder (NMOSD) were included. We employed an electronic record form on REDCap (https://www.project-redcap.org/) to collect study data. COVID-19 IgG was obtained as part of routine patient care. Repeat measurements were only available for 10 seropositive patients as the number of repeat measurements was determined by patient desire for repeat testing to evaluate durability of antibody response. Qualitative COVID-19 nucleocapsid protein specific IgG antibody testing was performed in house on an Abbott Architect platform (Abbott Park, IL). Data included was collected between January 2020 and November 2021, but follow-up data continues to be collected from the current cohort. Comorbidities included were cancer (prior or current), hypertension, cardiac disease (coronary artery disease or heart failure), chronic lung disease (chronic obstructive pulmonary disease, asthma, or sleep apnea), and diabetes mellitus (type 1 or type 2). Labs collected included white blood cell count, absolute lymphocyte count, vitamin D level, and IgG quantification in addition to COVID-19 IgG. A few patients in this cohort were included in our recent publication, but this focused on factors affecting COVID-19 severity and did not evaluate post-infection characteristics such as markers of humoral immunity (13). This study was approved by the Beth Israel Deaconess Medical Center IRB committee (Protocol# 2020P000343) and has therefore been performed in accordance with the ethical standards laid down in the 1964 Declaration of Helsinki and its later amendments.

Analyses were conducted using Microsoft Excel 2013 (Redmond, WA) for descriptive statistics and graphing and JMP Pro 16 statistical software (Cary, NC) for more complex statistics. Lab values, age, CNS demyelinating disease duration, Expanded Disability Status Scale (EDSS), and duration between events (e.g. infection and antibody collection) were treated as continuous variables with the remaining variables considered nominal. As the infection date for patients diagnosed by positive IgG was unknown given no prior COVID-19 related symptoms or positive PCR, infection date was considered the same as antibody collection date for those diagnosed by positive IgG obtained as part of routine care, and any analysis possibly affected by this assumption is highlighted in the results. The rare unknown variable in nominal categories was folded into the lower severity category or unknown (UNK), and the number of unknowns is detailed in the table/figure legends. There was no missing data for continuous variables. Hospitalization status, ICU admission, and method of COVID-19 infection diagnosis was obtained by treating physicians from either patient report or electronic medical record. DMTs were analyzed as individual treatments vs. none/other DMT with the exception of anti-CD20 monoclonal antibodies (i.e. rituximab and ocrelizumab), which were combined given similar mechanism of action. Univariate analysis was with Mann Whitney U test or ANOVA for continuous variables and Chi Square or Fisher Exact test for nominal variables. Univariate nominal logistic regression was run with target value of “positive.” Multivariate nominal logistic regression was run in a stepwise fashion based upon Akaike information criteria and mixed effects modeling. Significance was considered at *p* < 0.05 in all analyses.

## Results

We evaluated the demographic and clinical characteristics of 58 patients with available COVID-19 IgG testing, prior suspected or confirmed COVID-19 infection, and a known diagnosis of MS or NMOSD. We found an overall seropositive rate, or seroprevalence, of 42%. The characteristics of seronegative and seropositive patients are presented in Table 1. The majority of patients within the seropositive and seronegative groups, respectively, were female (62.5 vs. 70.6%), white (58.3 vs. 64.7%), not Hispanic (91.7 vs. 88.2%), never smokers (54.2 vs. 64.7%), not working (62.5 vs. 55.9%), and had RRMS (79.2 vs. 58.8%) (all *p* > 0.05). There was no significant difference in presence of major comorbidities (i.e. cancer, hypertension, lung disease, cardiac disease, and diabetes mellitus). The average duration of either NMOSD or MS was 15.6 ± 1.9 years in seropositive group and 13.6 ± 1.9 years in seronegative group (*p* > 0.05). Most recent EDSS (3.2 ± 0.5 in seropositive, 3.9 ± 0.5 in seronegative, *p* > 0.05) and vitamin D levels (43.2 ± 3.4 ng/nL in seropositive, 47.9 ± 3.0 ng/nL in seronegative, *p* > 0.05) were also similar.

**Table 1:** Demographics and clinical characteristics of COVID-19 IgG seropositive and seronegative patients with CNS demyelinating disorders and prior COVID-19 infection.

	**COVID-19 IgG antibody**	
	**+ (*n* = 24)**	**– (*n* = 34)**
**Prior COVID-19 vaccine** *[% (count)]*	25.0 (6)	44.1 (15)
**Method diagnosed** *[% (count)]*		
PCR Symptoms Antibody	58.3 (14) 20.8 (5) 20.8 (5)	76.5 (26) 23.5 (8) N/A
**Symptomatic** *[% (count)]*	75.0 (18)	91.2 (31)
**Hospitalized** *[% (count)]*	16.7 (4)	20.6 (7)
**Required ICU** *[% (count)]*	8.3 (2)	5.9 (2)
**Infection to antibody** *[average months (SEM)]*	2.3 (0.5)*	4.6 (0.7)
**Vaccinated prior to infection** *[% (count)]*	0.0 (0)	0.0 (0)
**Steroids within 1 month of infection** *[% (count)]*	4.2 (1)	8.8 (3)
**Gender** *[%(count)]*		
male female non-binary	33.3 (8) 62.5 (15) 4.2 (1)	29.4 (10) 70.6 (24) 0.0 (0)
**Age** *[average (SEM)]*	50.2 (2.8)	53.7 (2.1)
**Race** *[% (count)]*		
white black other/UNK	58.3 (14) 33.3 (8) 8.3 (2)	64.7 (22) 26.5 (9) 8.8 (3)
**Ethnicity** *[% (count)]*		
Hispanic	8.3 (2)	11.8 (4)
**Tobacco use** *[% (count)]*		
Ever user	45.8 (11)	35.3 (12)
**Employment** *[% (count)]*		
Working (PT+FT)	37.5 (9)	44.1 (15)
Retired/unemployed/disability/UNK	62.5 (15)	55.9 (19)
**Comorbidities** *[% (count)]*		
Cancer Hypertension Cardiac disease Chronic lung disease Diabetes mellitus	16.7 (4) 29.2 (7) 0 (0) 12.5 (3) 4.2 (1)	8.8 (3) 32.4 (11) 5.9 (2) 8.8 (3) 14.7 (5)
**MS type** *[% (count)]*		
RRMS SPMS/PPMS NMOSD	79.2 (19) 16.7 (4) 4.2 (1)	58.8 (20) 29.4 (10) 11.8 (4)
**Duration of MS /NMOSD** *[average years (SEM)]*	15.6 (1.9)	13.6 (1.6)
**EDSS** *[average score (SEM)]*	3.2 (0.5)	3.9 (0.5)
**Vitamin D level** *[average ng/nL (SEM)]*	43.2 (3.4)	47.9 (3.0)
**Current DMT** *(count)*		
none	3	4
rituximab/ocrelizumab	5*	19
natalizumab	6*	1
fingolimod dimethyl fumarate	1 5	2 2
teriflunomide glatiramer acetate interferon-beta	1 3 0	1 4 1

Patient characteristics, prior COVID-19 infection severity, and selected laboratory values are listed above, comparing COVID-19 IgG seropositive (middle column) vs. seronegative (right column) patients. There were significant differences between seropositive and seronegative patients by Chi Square or Mann Whitney U test with regards to duration between COVID-19 infection and antibody collection, anti-CD20 treatment (rituximab or ocrelizumab) vs. no/other DMT, and natalizumab vs. no/other DMT. Values are expressed as either percentages with counts in parenthesis or averages with standard error of the mean (SEM) in parenthesis. Working status was unknown for two seropositive and one seronegative patient. Significance of p < 0.05 is indicated by shading and ^*^. UNK, unknown; PT, part time; FT, full time; RRMS, relapsing remitting multiple sclerosis; SPMS, secondary progressive multiple sclerosis; PPMS, primary progressive multiple sclerosis; NMOSD, neuromyelitis optica spectrum disorder; EDSS, Expanded Disability Status Scale; DMT, disease modifying therapy; DMF, dimethyl fumarate.

With regards to the characteristics of prior COVID-19, the majority of infections were symptomatic (75.0% of seropositive, 91.2% of seronegative, *p* > 0.05) and were diagnosed by PCR as opposed to symptoms alone (58.3% of seropositive, 76.5% of seronegative, *p* > 0.05). Of note, eight patients who were diagnosed by symptoms alone were in the seronegative group. The majority of these infections occurred early in the pandemic, and testing was either not performed or not available. However, as our prior publication and many others include both suspected and confirmed COVID-19 cases, these patients were included in the dataset (9, 10, 13). Five patients were diagnosed by a positive COVID-19 antibody without any prior suspicion for infection. Although the time from suspected/confirmed COVID-19 infection to antibody collection was significantly longer in the seronegative group (2.3 ± 0.5 months in seropositive, 4.6 ± 0.7 months in seronegative, *p* < 0.01), this is likely biased by the patients diagnosed by antibody as this is no longer significant with their exclusion (*p* = 0.08). One patient in the seropositive group and three in the seronegative group received steroids within one month of COVID-19 infection although this was not found to impact seroprevalence (*p* > 0.05). In addition, some patients in each group received COVID-19 vaccination prior to antibody testing. As the vaccines received all target the spike protein, this has no effect on nucleocapsid specific IgG utilized in our study (14). There was no significant difference with respect to vaccination status, and no patients received a COVID-19 vaccine prior to infection.

**Figure 1:**
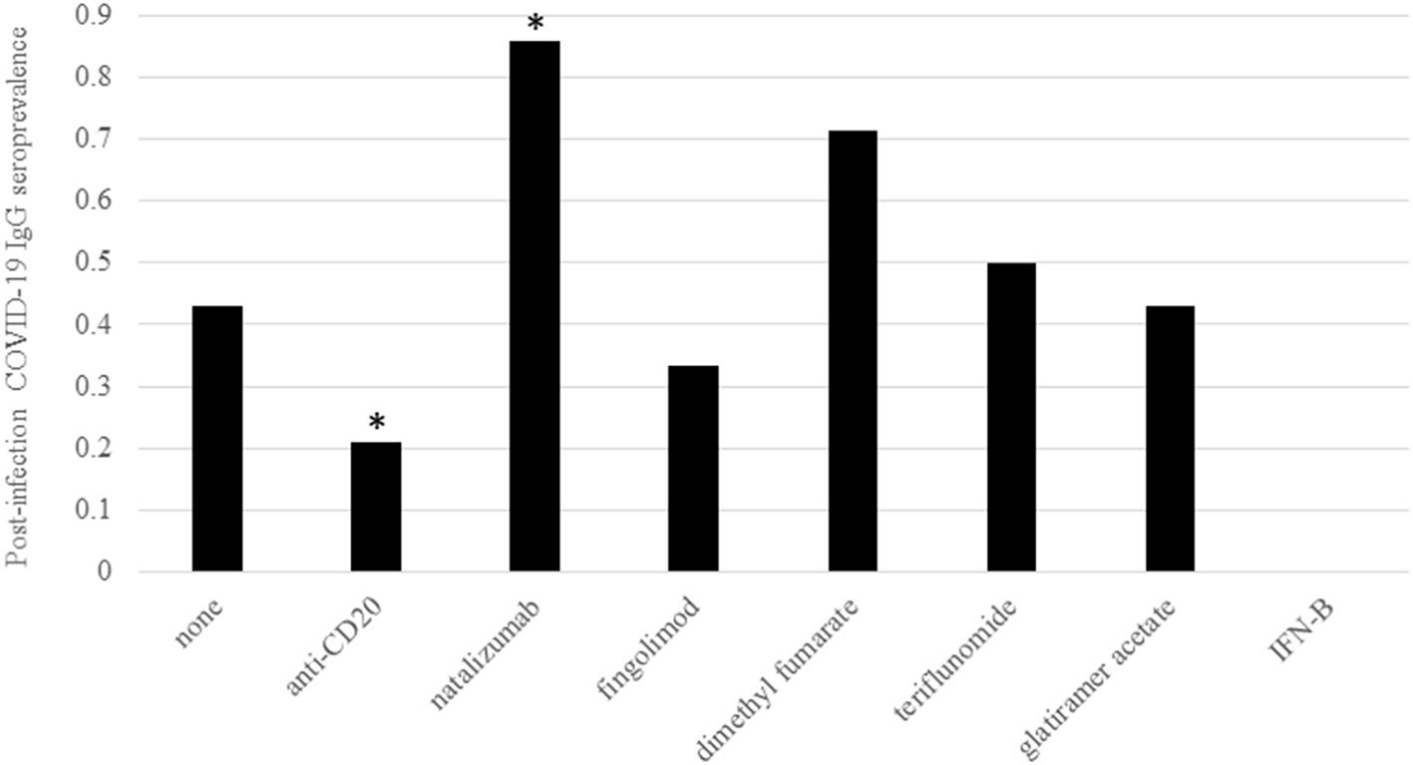
Post-infection COVID-19 IgG prevalence by DMT. Prevalence of post-infection COVID-19 IgG antibody positivity was grouped by DMT type. All DMTs were considered individual groups with the exception of the anti-CD20 monoclonal antibodies rituximab and ocrelizumab. DMT type is indicated on the X axis and COVID-19 IgG seroprevalence is on the Y axis. As shown in Table 1, there was a significantly lower rate of seroprevalence in patients on anti-CD20 treatments and significantly higher rate of seroprevalence in patients on natalizumab via Chi Square or Fisher Exact test compared to patients on no/other DMTs. The number of patients within the seropositive group on each treatment were none = 3, anti-CD20 = 5, natalizumab = 6, fingolimod = 1, dimethyl fumarate = 5, teriflunomide = 1, glatiramer acetate = 3, and interferon-beta = 0. The number of patients within the seronegative group on each treatment were none = 4, anti-CD20 = 19, natalizumab = 1, fingolimod = 2, dimethyl fumarate = 2, teriflunomide = 1, glatiramer acetate = 4, and interferon-beta = 1. Significance (*p* < 0.05) is indicated by *.

As expected, DMT type significantly impacted post-infection COVID-19 IgG seroprevalence (Table 1; Figure 1). Only five of 24 patients on rituximab or ocrelizumab formed antibodies to COVID-19 after infection (*p* = 0.01 for anti-CD20 monoclonal antibody vs. none/other DMT). Although limited by small sample size, there were no significant differences between seronegative and seropositive patients on rituximab or ocrelizumab with regards to average months between COVID-19 infection and antibody collection, months between most recent anti-CD20 infusion and infection or antibody, duration of anti-CD20 treatment, number of infections that were symptomatic, or most recent labs (i.e. white blood cell count, absolute lymphocyte count, IgG quantification) (*p* > 0.05, Table 2). Additionally, six of seven patients on natalizumab developed COVID-19 nucleocapsid protein IgG antibodies after infection (*p* = 0.02 for natalizumab vs. none/other DMT, Table 1; Figure 1). One of the six seropositive patients had an asymptomatic infection, and none of the patients on natalizumab required hospitalization for COVID-19 infection or received high dose IV steroids within a month of infection. The range of months between infection to antibody was similar (1–3 months in seropositive, 3 months in seronegative).

**Table 2:** Post-infection seropositive and seronegative characteristics of patients on anti-CD20 monoclonal antibodies.

	**COVID-19 IgG antibody**	
	**+ (*n* = 5)**	**– (*n* = 19)**
**Symptomatic COVID-19 infection** *[%(count)]*	60.0 (3)	94.7 (18)
**Infection to antibody** *[average months (SEM)]*	1.6 (0.9)	2.9 (0.4)
**Last infusion to antibody** *[average months (SEM)]*	7.6 (1.5)	7.1 (0.6)
**Last infusion to infection** *[average months (SEM)]*	4.3 (2.4)	5.0 (0.8)
**Duration of treatment** *[average months (SEM)]*	30.4 (8.9)	44.7 (7.0)
**Most recent WBC** *[average K/μL (SEM)]*	6.9 (0.4)	6.6 (0.6)
**Most recent ALC** *[average K/μL (SEM)]*	1240 (103)	1661 (184)
**Most recent IgG level** *[average mg/dL (SEM)]*	1134 (129)	887 (60)
**Current DMT** *(count)*		
rituximab	1	9
ocrelizumab	4	10

*Characteristics of COVID-19 infection, durations between most recent anti-CD20 antibody infusion and infection/antibody collection, and selected laboratory values are listed above, comparing COVID-19 IgG seropositive (middle column) vs. seronegative (right column) patients on anti-CD20 agents. Despite the low rate of seropositivity in this group compared to no/other DMTs, we found no significant differences between seropositive and seronegative patients by Chi Square or Mann Whitney U test. Values are expressed as either percentages with counts in parenthesis or averages with standard error of the mean (SEM) in parenthesis. Significance was considered at p < 0.05. WBC, white blood cell count; ALC, absolute lymphocyte count; DMT, disease modifying therapy*.

**Figure 2:**
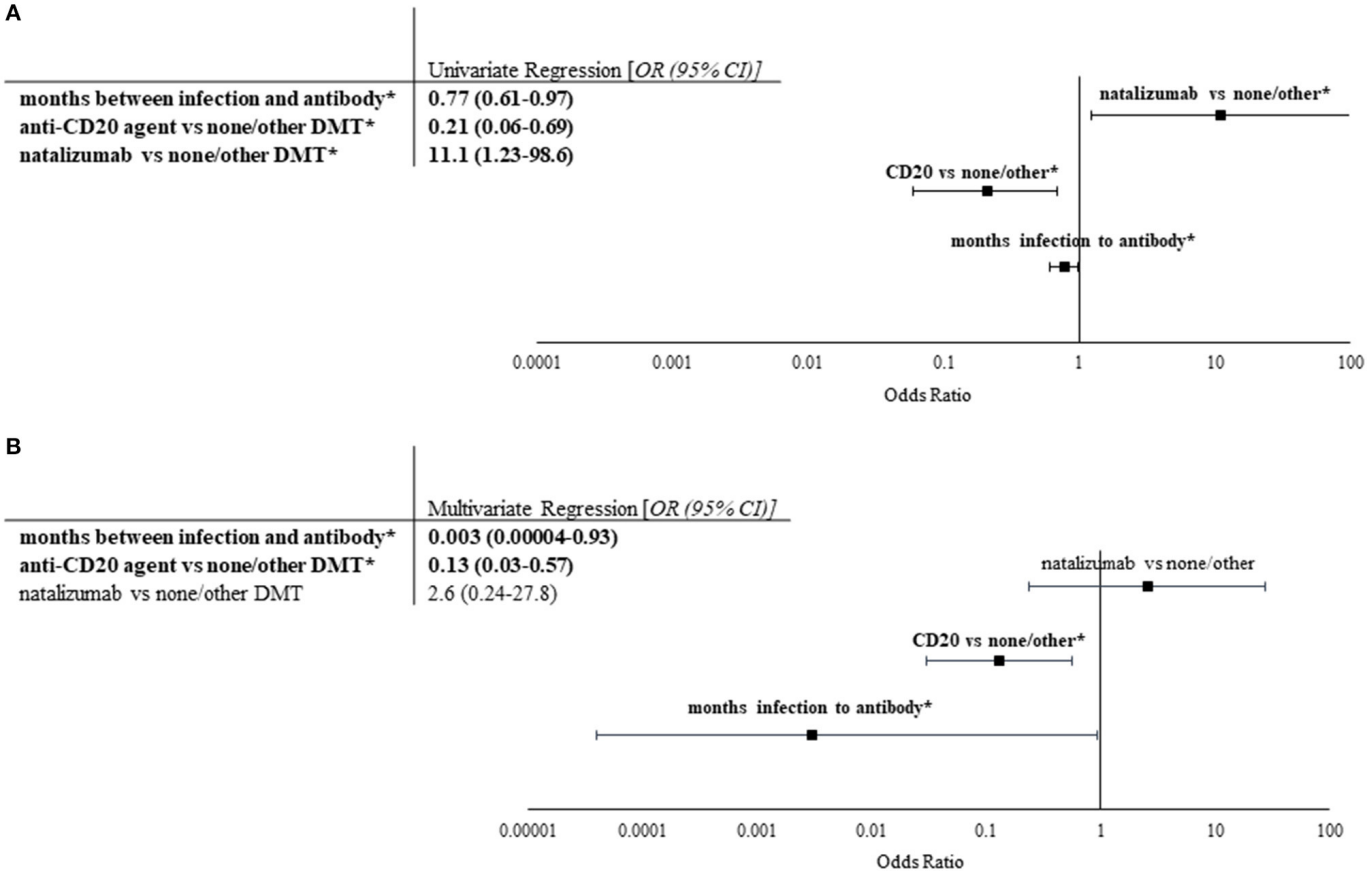
Univariate and multivariate analyses of factors impacting seropositivity. Univariate and multivariate nominal logistic regression analyses were performed on patient characteristics, prior COVID-19 infection severity, and selected lab values based upon COVID-19 IgG antibody values (*n* = 24 for seropositive, *n* = 34 for seronegative). **(A)** Univariate logistic regression was performed on characteristics of cohort. Odds ratios with 95% confidence intervals of all significant factors are listed in the table to the left. Significant correlations are indicated by bold font and *. Longer duration between COVID-19 infection and antibody collection as well as use of anti-CD20 treatment (rituximab or ocrelizumab) compared to no/other DMT were found to be predictive of negative post-infection COVID-19 IgG, whereas use of natalizumab vs. no/other DMT was found to be predictive of positive COVID-19 IgG. **(B)** Stepwise multivariate logistic regression utilizing Akaike information criteria was performed using significant factors in univariate analysis. Odds ratios with 95% confidence intervals of all factors evaluated are shown in the table (left) and figure (right). Only longer duration between COVID-19 infection and antibody collection and use of anti-CD20 treatment were found to independently decrease likelihood of seropositivity.

Univariate and multivariate nominal logistic regression was utilized to evaluate dependent and independent predictive factors for seropositivity. Time between infection and antibody collection (OR 0.77, 95% CI 0.61–0.97), anti-CD20 treatment vs. other/none (OR 0.21, 95% CI 0.06–0.69), and natalizumab vs. other/none (OR 11.1, 95% CI 1.23–98.6) were significant contributors in univariate analysis (Figure 2). However, only time between infection and antibody collection (OR 0.003, 95% CI 0.00004–0.93, *p* < 0.01) and anti-CD20 treatment vs. other/none (OR 0.13, 95% CI 0.03–0.57, *p* < 0.01) were found to be independent predictors of seropositivity (Figure 2).

Multiple post-infection COVID-19 antibody measurements were available for ten seropositive patients. Five demonstrated continued antibody positivity for 3–9 months after COVID-19 infection (Table 3). Two of these patients were on natalizumab, and three were on dimethyl fumarate. Additionally, five patients within the cohort converted from seropositive to seronegative between 3 and 8 months after mild COVID-19 infection (Table 4). Two patients were on ocrelizumab; one was on natalizumab; one was on dimethyl fumarate; and, one was not on a DMT. As these small subsets are biased by data availability, it is difficult to assess whether differences in demographic characteristics, such as age or race, contributed to sustained seropositivity or lack thereof.

**Table 3:** Patient characteristics with persistently positive COVID-19 IgG.

**Method infection diagnosis**	**Hospitalzation**	**Age**	**Gender**	**Race**	**Ethnicity**	**Smoking**	**Work status**	**MS type**	**Current DMT**	**Months between infection and first positive antibody**	**Months between infection and last positive antibody**
antibody	no	42	F	black	not Hispanic	former	no	RRMS	natalizumab	0	3
PCR	no	77	F	black	not Hispanic	never	no	RRMS	dimethyl fumarate	3	3.5
symptoms	no	53	non-binary	white	not Hispanic	never	full time	RRMS	natalizumab	2	5
PCR	no	41	F	other	not Hispanic	never	full time	RRMS	dimethyl fumarate	0	6
PCR	no	40	M	black	not Hispanic	former	full time	RRMS	dimethyl fumarate	3	9

*Prior COVID-19 infection severity, patient characteristics, and durations between infection and antibody collection are listed above for patients found to have a persistently positive COVID-19 IgG over time. Multiple measurements of post-infection COVID-19 antibody were only available for a small subset of patients. Only 5 of the 10 seropositive patients with multiple measurements were found to remain persistently positive. This included 2 RRMS patients on natalizumab and 3 RRMS patients on dimethyl fumarate with an antibody positivity ranging from 3 to 9 months post-infection. No patients in this group required hospitalization for COVID-19 infection, and only one patient had an asymptomatic antibody-diagnosed infection. Statistical analysis was not performed on differences between these patients given the small number. F, female; M, male; RRMS, relapsing remitting multiple sclerosis; DMT, disease modifying therapy; DMF, dimethyl fumarate*.

**Table 4:** Characteristics of patients who converted from seropositive to seronegative.

**Method infection diagnosis**	**Hospitalzation**	**Age**	**Gender**	**Race**	**Ethnicity**	**Smoking**	**Work status**	**MS type**	**Current DMT**	**Months between infection and last positive antibody**	**Months between infection and seroreversion**
antibody	no	34	F	black	not Hispanic	never	no	RRMS	ocrelizumab	0	6
PCR	no	42	F	black	not Hispanic	never	full time	RRMS	none	0	3
PCR	no	46	M	white	not Hispanic	never	no	RRMS	natalizumab	1	8
PCR	no	31	M	black	Hispanic	current	UNK	RRMS	ocrelizumab	2	8
symptoms	no	47	F	white	not Hispanic	never	no	RRMS	dimethyl fumarate	3	8

*Prior COVID-19 infection severity, patient characteristics, and durations between infection and antibody collection are listed above for patients found to convert from antibody positive to negative over time. Multiple measurements of post-infection COVID-19 antibody were only available for a small subset of patients. Only 5 of the 10 seropositive patients with multiple measurements converted to seronegative. This subset of all RRMS patients included two on ocrelizumab, one on dimethyl fumarate, one on natalizumab, and one not on a DMT. Loss of seropositivity occurred between 3 and 8 months post-infection. No patients required hospitalization in this group. Statistical analysis was not performed on differences between these patients given the small number. F, female; M, male; RRMS, relapsing remitting multiple sclerosis; DMT, disease modifying therapy; UNK, unknown; DMF, dimethyl fumarate*.

## Discussion

Herein, we demonstrate a seropositivity rate (or seroprevalence) of 42% in patients with MS and NMOSD after suspected or confirmed COVID-19 infection. This is similar to most case series and small cohort studies of patients with CNS demyelinating disorders around the world (5–8). However, it is drastically lower than the >90% seropositivity within the first 6 months post-infection in the general population and the 76–80% observed in two recent international cohorts (1, 3, 9, 10, 15). As we observed a lower seropositivity rate in patients not on a DMT, we postulate factors independent of immunosuppression are at play, such as significant disability, prior exposure to immunosuppressants, and possibly altered humoral immunity secondary to inherent genetic and/or environmental factors associated with MS (16, 17). We suspect our findings are different from other MS cohorts due to significantly younger age of patients in these cohorts, multiple/different COVID-19 antibody assays utilized, and significantly higher percentage of severe COVID-19 infection in one cohort (9, 10). Our findings show that antibody positivity decreases over time as would be expected and that use of B cell depleting agents, like rituximab and ocrelizumab, are independent predictors of a negative post-infection antibody.

We found no impact of COVID-19 severity, age, sex, presence of major comoribities, or PCR confirmation of infection similar to other cohorts of MS patients (11, 18, 19). Not unexpectedly, we did observe a decreased likelihood of seropositivity with a longer duration between infection and antibody collection. This was impacted significantly by the individuals diagnosed by positive antibody as this was no longer significant with their removal. It is possible that subtle effects were missed due to the small size of our cohort. For example, increasing age has been associated with higher rates of seronegativity with a sample size of 3.2 million (20). However, our primary objective was to evaluate differences related to DMT use, and we anticipate larger studies and/or increased size of our cohort will illuminate these subtleties in time.

Although most studies have found little impact of DMT on COVID-19 severity (13, 21–24), this is not the case for post-infection humoral immunity. Patients on ocrelizumab or rituximab in our cohort were less likely to form COVID-19 IgG antibodies independent of any other factor. This was expected given the critical role of B cells in humoral immunity and the increasing suspicion for more severe COVID-19 infection in patients on anti-CD20 treatments (25–29). An impact of anti-CD20 monoclonal antibodies on post-infection seropositivity has been seen in other cohorts (5–7, 9–11), and parallels the blunted humoral response to non-live vaccines in patients on ocrelizumab noted in the VELOCE study (30). We anticipated a similar effect with the lymphocyte sequestering agent fingolimod, but there were only three patients on fingolimod in our cohort. Without a larger sample of post-infection or post-vaccination COVID-19 antibody data in patients on fingolimod, the impact of fingolimod in this context remains unclear (7, 31, 32).

Unexpectedly, natalizumab was associated with a higher seropositivity rate in univariate analyses, although was not an independent risk factor in multivariate regression. In a small Italian study of fifty MS patients on natalizumab, the rate of seropositivity was double the local general population at 16%, but interestingly only three patients reported symptoms suggestive of a prior COVID-19 infection (32). Natalizumab is a selective adhesion molecule inhibitor that decreases specific immune cell trafficking through the blood brain barrier to the central nervous system. We hypothesize that this population is more similar to the general population with regards to systemic immune competency as compared to other DMTs, but it is also possible that natalizumab is associated with an increased rate of minimally symptomatic or asymptomatic infections (33). Future studies will hopefully shed further light on this association.

As studies utilizing quantitative COVID-19 serology have demonstrated at least some COVID-19 antibody titer post-infection present in most MS patients after infection, the impact of DMT type is likely on both intensity and durability of antibody response (11). Studies in the general population have found post-infection COVID-19 antibodies to be present for months although the rate of decline remains controversial with some data suggesting gradual decline over weeks (2–4, 15, 34). However, few studies have been able to evaluate durability of antibody response in MS patients. We were able to demonstrate continued antibody positivity in five patients with seropositive duration as long as 9 months after infection. Conversely, we found seroreversion in five patients with some negative as early as 3 months after infection. As we collect additional data, we hope to better delineate the duration of humoral immunity post-infection.

The clinical significance of decreased post-infection antibodies could imply more than impaired humoral immunity. Immunologic studies suggest COVID-19 antibody titers parallels COVID-19 specific cellular immunity (35–37). Peak nucleocapsid antibody response correlates strongly with T cell response (36, 38). Similar to memory B cell formation in convalescent COVID-19, stem cell like memory T cells have been found in seropositive patients as well as seronegative patients although at a much lower rate (35). Even the COVID-19 vaccine BNT162b1 trials demonstrated an increased COVID-19 specific T cell response post-vaccination, which also correlated with antibody titers (39). Unfortunately, the cellular immune response in the MS community has not been widely studied. However, two studies of patients on ocrelizumab demonstrate a higher T cell response than anticipated from humoral response after vaccination and infection (40, 41). Given the varying mechanisms of action utilized by DMTs, it is difficult to otherwise predict the degree of humoral and cellular immunity correlation in our cohort.

Our study is not without limitations. Our analysis is limited by small sample size, especially when querying individual DMTs. Geographic location, even within the United States, also has demonstrated effect on seroprevalence, which we are unable to assess in our Boston-based cohort (19, 20, 42). There is also unavoidable variability introduced by fluctuating seroprevalence over time (43). We did not have data on COVID-19 treatments received during acute illness. As the COVID-19 antibody data was collected as part of routine care, variability in timing of antibody collection was introduced and only nucleocapsid protein antibody testing was utilized. This also impacted the number of patients with repeat antibody measurements as many patients opted not to obtain consecutive measurements.

In conclusion, we present data from 58 patients with MS or NMOSD and prior COVID-19 infection, showing an overall lower seropositivity rate than observed in the general population that was further decreased by use of anti-CD20 monoclonal antibodies and longer time from infection to antibody collection. Importantly, we demonstrate some humoral immunity with almost all included DMTs, lasting as long as 9 months post-infection. Although humoral immunity is thought to parallel both neutralizing antibody activity and T cell response, we have reservations that this generalization can be made in the MS population given the varied mechanisms of action utilized by DMTs. As the pandemic continues, we hope that further studies will build upon this to further delineate the DMT-specific humoral and cellular immunity both post-infection and post-vaccination to better guide patient care.

## Data Availability Statement

The raw data supporting the conclusions of this article will be made available by the authors, without undue reservation.

## Ethics Statement

The studies involving human participants were reviewed and approved by Beth Israel Deaconess Medical Center IRB Committee (Protocol# 2020P000343). Written informed consent for participation was not required for this study in accordance with the national legislation and the institutional requirements.

## Author Contributions

KM performed drafting/revision of manuscript and analysis/interpretation of data. JS designed study, performed drafting/revision of manuscript, and assisted with acquisition of data and data analysis. Remaining authors played a major role in data acquisition. All authors contributed to the article and approved the submitted version.

## Funding

Outside of this work, JS has grant funding from National MS Society, Biogen, and Genentech and has consulted for Biogen, Genentech, Teva, Banner, Sanofi, and Cellgene.

## Conflict of Interest

The authors declare that the research was conducted in the absence of any commercial or financial relationships that could be construed as a potential conflict of interest.

## Publisher's Note

All claims expressed in this article are solely those of the authors and do not necessarily represent those of their affiliated organizations, or those of the publisher, the editors and the reviewers. Any product that may be evaluated in this article, or claim that may be made by its manufacturer, is not guaranteed or endorsed by the publisher.
